# Mosquito larvicidal activity of *Cassia tora* seed extract and its key anthraquinones aurantio-obtusin and obtusin

**DOI:** 10.1186/s13071-017-2512-y

**Published:** 2017-11-10

**Authors:** Valentine C. Mbatchou, David P. Tchouassi, Rita A. Dickson, Kofi Annan, Abraham Y. Mensah, Isaac K. Amponsah, Julia W. Jacob, Xavier Cheseto, Solomon Habtemariam, Baldwyn Torto

**Affiliations:** 1 Department of Applied Chemistry and Biochemistry, University for Development Studies, Navrongo Campus, Navrongo, Ghana; 2 Department of Pharmacognosy, Faculty of Pharmacy and Pharmaceutical Sciences, Kwame Nkrumah University of Science and Technology, Kumasi, Ghana; 3 International Centre of Insect Physiology and Ecology, P.O. Box 30772, Nairobi, Kenya; 4 Pharmacognosy Research Laboratories and Herbal Analysis Services UK, University of Greenwich, Chatham-Maritime, Kent, ME4 4TB UK; 5 Department of Zoology and Entomology, University of Pretoria, Pretoria, South Africa

**Keywords:** *Cassia tora*, Aurantio-obtusin, Obtusin, Anthraquinone, Mosquito larvicidal activity, *Anopheles gambiae S.S*

## Abstract

**Background:**

The edible and medicinal leguminous plant *Cassia tora* L. (*Fabaceae*) is known to possess insecticidal properties against a wide range of plant-feeding insects. However, the bioactivity of extracts of this plant and their constituents against vectors of medical importance has been largely unexplored. We investigated the mosquito larvicidal activity of the seed extract and its major anthraquinones against larvae of the African malaria vector *Anopheles gambiae* (*s.s*.).

**Methods:**

Third-fourth instar larval mortality was observed after 24, 48, 72 and 96 h of exposure to varying doses of the extracts, and two anthraquinones isolates identified using liquid chromatography- quadrupole time of flight mass spectrometry (LC-QtoF-MS). The mosquito larval mortality was evaluated relative to the natural insecticide azadirachtin.

**Results:**

Fractionation of the crude extract decreased mosquito larvicidal activity, however, larvicidal activity increased with increasing dose of the treatment and exposure time. The known anthraquinones aurantio-obtusin and obtusin were identified as key larvicidal compounds. Aurantio-obtusin and obtusin, exhibited similar toxicity to larvae of *A. gambiae* (*s.s*.) with LD_50_ values of 10 and 10.2 ppm, respectively. However, the two anthraquinones were four- and ~ six-fold less potent than that of the crude seed extract and azadirachtin, which had comparable LD_50_ values of 2.5 and 1.7 ppm, respectively.

**Conclusion:**

Both aurantio-obtusin and obtusin showed mosquito larvicidal activity which were comparable to their respective fractions although they were less potent relative to the crude extract and azadirachtin. Further studies need to be conducted on *C. tora* for its exploitation as a potential eco-friendly tool in mosquito larval source reduction.

## Background

Mosquito control has become a global health priority, owing to their vectoring role of pathogens of many diseases such as malaria, dengue, yellow fever, Zika, West Nile, affecting a significant proportion of the world population. These diseases contribute significantly to the estimated 17% of the global vector-borne disease burden of all infectious diseases, accounting for >1 billion new cases and >1 million deaths annually [1]. Current control strategies against these diseases mainly targeting vector populations overwhelmingly rely on the classic integrated vector management (IVM). This involves entomological surveillance, community involvement through environmental management to eliminate breeding sites, and application of pyrethroid insecticides against adults and *Bacillus thuringiensis* var. *israelensis* (Bti) against larvae [2]. Besides the growing concerns related to sustainability of these measures, there is the challenge of widespread evolution of resistance in mosquito populations to insecticides and even the biopesticides [3–5].

The need for safe and ecologically friendly tools has spurred interest in the use of botanicals in mosquito larval control commonly referred to as larval source management [6, 7]. Controlling mosquitoes as larvae seems more feasible than adults as they are relatively immobile and often readily accessible compared to adults which can disperse and change their habitat to avoid control measures [7]. The plant *Azadirachta indica* (Meliaceae), commonly referred to as neem, is a classic botanical whose insecticidal activity of the crude or derived products have been evaluated against many insects including vectors of medical importance [3, 6, 8]. In addition, various plant-derived compounds for controlling medically and veterinary important insect vectors have been highlighted [9, 10]. On the other hand, to date, most of the work carried out on *Cassia tora* L. (Fabaceae) an edible and medicinal leguminous plant, widely distributed in farmlands in Central Africa [11], has been on its antifeedant and repellent activities on a range of crop pests including *Zonocerus variegatus* and the cowpea weevil [11, 12]. The plant has also been found to suppress the spread of the invasive weed *Centrosema pubescens* [12]. However, *C. tora* which produces large quantities of fruits has an unexplored potential for control of insect pests such as medical disease vectors. In this study, we aimed to assess the potential of the crude extract, its solvent fractions and two major anthraquinones against larvae of the malaria vector *Anopheles gambiae* (*s.s*.).

## Methods

### Insects

For bioassays, we used third-fourth-instar larvae of *A. gambiae* (Mbita strain) obtained from a colony maintained at the insectary of the Duduville Campus of the International Centre of Insect Physiology and Ecology (*icipe*) in Nairobi. The strain originally from Mbita Point, western Kenya has been maintained in colony reared under laboratory conditions for over 20 years. The mosquito larvae were fed on Tetramin fish food (Melle, Germany) and the rearing conditions were maintained at a mean temperature of 31 °C and relative humidity of 52% RH during the day while at night the mean temperature and relative humidity were 24 °C and 72% RH, respectively (12 h light and 12 h dark).

### Plant material

Pods containing *C. tora* seeds were randomly harvested from trees near Kramah quarters, Kumba (4°38′N, 9°26.4′E), South-West Region, Cameroon in March 2013. They were identified by botanists at the Department of Applied Biology (University for Development Studies, Ghana) where a voucher specimen (AB/4/160/13) has been deposited.

### Isolation of aurantio-obtusin and obtusin

**Fig. 1:**
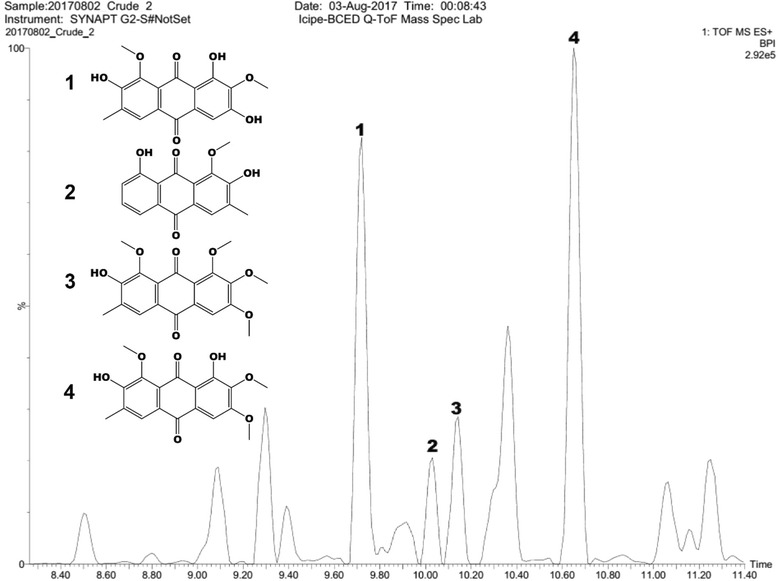
LC-QTOF-MS representative total ion chromatogram showing compounds identified in the seed extract of *C. tora*

Isolation of the two anthraquinones aurantio-obtusin and obtusin was carried out as previously described with minor modifications [13]. Briefly, the seed-pods of *C. tora* were opened by hand and the seeds air-dried at room temperature (25 °C) for 1 week, and ground into powder with the aid of mortar and pestle. The powdered seeds (5 kg) were defatted by Soxhlet extraction using petroleum ether 1 *v*/*w* (Fisher Scientific Co. Pittsburgh, Pennsylvania, USA) for 12 h and then extracted in ethyl acetate 5 *v*/*w* (Merck KGaA, Darmstadt, Germany) until exhaustion (48 h) to yield 25.5 g of the crude extract. The ethyl acetate extract (25.5 g) was loaded onto a silica gel (70−150 Mesh ASTM) glass column of diameter 35 mm (400 mm long) and eluted with solvents of increasing polarity using petroleum ether and ethyl acetate (from 100% petroleum ether with increasing amounts of ethyl acetate to 100% of it to yield 125 fractions). The eluates were then concentrated under reduced pressure on a rotary evaporator (Laborata 4000; Heidolph instruments GmbH & Co. KG, Germany) at 40 °C and bulked into five fractions based on their thin layer chromatography profiles. The plates were visualized under UV-lamp at 254 and 365 nm and sprayed with ethanolic KOH. Repeated column chromatography on silica gel of fractions 3 and 4 using gradient mixtures of petroleum ether and ethyl acetate afforded aurantio-obtusin **1** (1.95 g) and obtusin **4** (0. 72 g) (Fig. 1) from a 10:90% petroleum ether and ethyl acetate fraction. The identities of aurantio-obtusin **1** and obtusin **4** were confirmed by co-injection of the isolated samples with the crude seed extract and analysed by liquid chromatography-quadrupole time of flight-mass spectrometry (LC-QtoF-MS).

### LC-QTOF-MS analysis of *C. tora* seed extract

The seed extract of *C. tora* (10 mg), was dissolved in 1 ml [5:95:water (0.01% formic acid)], acetonitrile, LC-MS grade CHROMASOLV, Sigma-Aldrich, St. Luis, MO, USA), vortexed for 30 s, and centrifuged at 14,000× *rpm* for 5 min, after which 0.2 μl of the supernatant was analysed on a Waters ACQUITY UPLC I-class system (Waters Corp., Milford, MA, USA) fitted with an ACE C18 column, 4.6 × 250 mm × 4.6 μm (Scotland, UK) with a heater turned off and an autosampler tray cooled to 5 °C. Mobile phases of water A and acetonitrile B, each with 0.01% formic acid was employed. The following gradient was used 0 min, 5% B; 0–3 min, 5–30% B; 3–6 min, 30% B; 6–7.5 min, 30–80% B; 7.5–10.5 min, 80% B; 10.5–13.0 min, 80–100% B, 13–18 min, 100% B; 18–20 min, 100–5% B; 20–22 min, 5% B, run time 25 min. The flow rate was held constant at 0.3 ml/min.

The UPLC system was interfaced by electrospray ionization to a Waters Synapt G2-Si QTOF-MS operated in full scan MSE in positive mode. Data were acquired in resolution mode over the m/z range 100–1500 with a scan time of 1 s using a capillary voltage of 0.5 kV, sampling cone voltage of 40 V, source temperature of 100 °C and desolvation temperature of 350 °C. The nitrogen desolvation flow rate was 500 l/h. For the high-energy scan function, a collision energy ramp of 25–45 eV was applied in the T-wave collision cell using ultrahigh purity argon (≥ 99.999%) as the collision gas. A continuous lock spray reference compound (leucine enkephalin; [M + H] + = 556.2766) was sampled at 10 s intervals for centroid data mass correction. The mass spectrometer was calibrated across the 50–2000 Da mass range using a 0.5 mM sodium formate solution prepared in 90:10 propan-2-ol:water. MassLynx version 4.1 SCN 712 (Waters Corp., Milford, MA, USA) was used for data acquisition and processing. The elemental composition was generated for every analyte. Potential assignments were calculated using the monoisotopic masses with specifications of a tolerance of 10 ppm deviation and both odd- and even-electron states possible. The number and types of expected atoms were set as follows: carbons ≤ 50; hydrogens ≤ 100; oxygens ≤ 50; nitrogens ≤ 10; chlorines ≤ 10; sulfurs ≤ 10. The empirical formula generated was used to predict structures that were proposed based on the online database (METLIN, ChemSpider and ChemCalc, CSI:Fingerid), fragmentation pattern and literature [14–17].

### Mosquito larvicidal assay

Mosquito larvicidal assay was carried out following standard procedure described by WHO [18] and adopted by Ndung’u et al. [19–21] with an exception of the solvents used, dimethyl sulfoxide (DMSO, 99.9%, Sigma-Aldrich), instead of acetone. Briefly, 1 ml standard *w*/*v* of each test material or treatment in DMSO was made up to 20 ml with distilled water in 100 ml beakers in three replicates. Azadirachtin, a potent anti-insect naturally occurring limonoid [22], previously isolated and characterized in our laboratory from neem *Azadirachta indica* [20] and used as a positive control was similarly prepared in DMSO (1 ml). Twenty late third-fourthinstar larvae each were transferred into the test and control solutions, and larval mortality was monitored and recorded for up to 96 h. Dead larvae were removed from each treatment daily (after 24 h). The room temperature was maintained at 25–27 °C and larvae in each treatment were fed daily with approximately 1 mg of Tetramin fish food (Melle, Germany).

For the assays, we prepared a stock solution of 10 mg/ml by dissolving 40 mg of crude extract, fractions 3 and 4, and compound **1** in 4 ml of dimethyl sulfoxide (DMSO) and 2.5 mg of compound **4** in 0.25 ml DMSO (due to limited material available). From the stock solution, five concentrations of 0.1, 0.01, 0.005, 0.0025 and 0.001 mg/l (corresponding to 100, 10, 5, 2.5 and 1 ppm, respectively) for the crude extract, fractions 3 and 4 and compound **1**, and only three concentrations of 0.01, 0.0025 and 0.001 mg/ml (corresponding to 5, 2.5 and 1 ppm, respectively) for compound **4**.

### Statistical analysis

The mortality data after 96 h were subjected to Probit analysis to determine the relative toxicity of each of the compounds and controls to the larvae. The dose used was log-transformed and data subjected to a generalized linear model (GLM) binomial regression model with the link probit function with the binomial response variables (no. dead/no. alive). We compared the differences in dose responses for each compound and controls by estimating LD_50_ and LD_90_, and the corresponding 95% confidence intervals. Also, the number of dead larvae in each replicate of a treatment were converted into proportions and analyzed by ANOVA after arcsine-transformation to normalize the distribution. Mean mortalities induced by each treatment and dose were compared by Tukey’s HSD test. All analyses were performed in R version 3.3.1 (R Development Core Team) at α = 0.05 level of significance.

## Results

### Chemical analysis

LC-QtoF-MS analysis revealed that anthraquinones dominated the seed extract of *C. tora*; four of which were identified as aurantio-obtusin, obtusiolin, chryso-obtusin and obtusin (Fig. 1, Table 1). Aurantio-obtusin and obtusin were isolated from the bioactive fractions 3 and 4, respectively, and confirmed by co-injection with the crude extract and analysed by LC-QtoF-MS.

**Table 1:** Retention time and major fragment ions of compounds identified in the seed extract of *C. tora*

Peak no.	Retention time (min)	Compound name	[M + H]^+^	Major fragments	Reference
1	9.72	Aurantio-obtusin	331.0824	316.0610, 298.0490, 288.0645, 270.0531, 253.0491, 242.0589	[14–17]
2	10.03	Obtusiolin	285.0766	270.0522, 255.0648, 237.0510, 196.0431, 177.1118, 133.0861	[14–17]
3	10.14	Chryso-obtusin	359.1142	326.0758, 270.3160, 268.0780, 211.0748, 177.1149, 133.0853	[14–17]
4	10.65	Obtusin	345.0931	330.0735, 312.0622, 282.0526, 254.0513, 238.0612, 154.0484	[14–17]

### Mosquito larvicidal activity

**Fig. 2:**
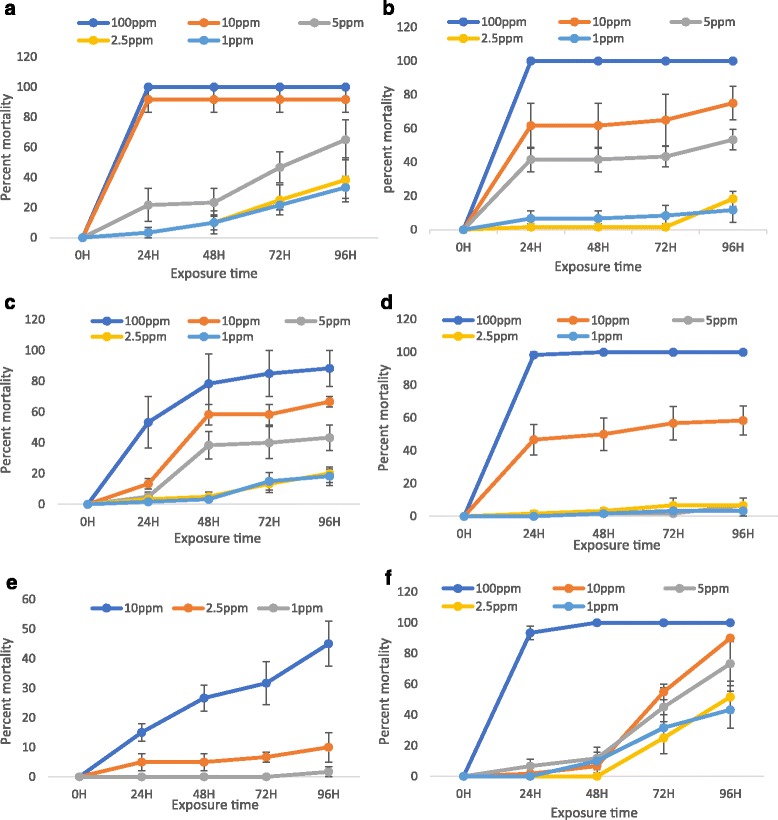
Mean percent mortalities of *A. gambiae* larvae induced by different doses of the treatments. **a** Crude *C. tora* extract. **b** Fraction 3. **c** Fraction 4. **d** Aurantio-obtusin. **e** Obtusin. **f** Azadirachtin

Fractionation of the extract decreased mosquito larvicidal activity, although irrespective of the treatment, larvicidal activity increased with increasing dose and time of exposure of the treatment (Table 2, Fig. 2). There was a significant difference in larval mortality for the different compounds tested at different doses (*F*_(6, 27)_ = 20.77, *P* < 0.001). Fractions 3 and 4 were ~ two- to four-fold less potent than the crude extract and the positive control azadirachtin, as indicated by their LD_50_ values (5 ppm, 95% CI: 0.004–0.006 for fraction 3; 7 ppm, 95% CI: 0.005–0.01 for fraction 4) and exposure time (Fig. 2). Likewise, aurantio-obtusin (**1)** and obtusin **(4)**, had LD_50_ values of 10 ppm (95% CI: 0.02–0.05) and 10.2 ppm (95% CI: 0.02–0.12), respectively, and were four- and ~six-fold less potent than the crude seed extract and the positive control azadirachtin respectively, which had LD_50_ values of 2.5 (95% CI: 0.001–0.003) and 1.69 ppm (95% CI: 0.008–0.02), respectively and exposure time (Fig. 2; Table 2). Interestingly, except for fraction 4 whose LD_90_ value was 90 (95% CI: 0.043–0.185), the LD_90_ values of the other treatments were not significantly different from that of the positive control.

**Table 2:** Mosquito larvicidal activity (percent mortality ± SE) of the crude and active fractions of *C. tora*, and pure compounds against third and fourth-instars of *A. gambiae*

	1 ppm	2.5 ppm	5 ppm	10 ppm	100 ppm	LD50 (95% CI)	LD90 (95% CI)
Crude extract	33.3 ± 7.3 Ac	38.3 ± 14.5 Ac	65 ± 13.2 Abc	91.7 ± 8.3Aab	100 ± 0 Aa	2.5 (0.001–0.003)A	10.36 (0.009–0.021) A
Fraction 3	11.7 ± 7.3 Bc	18.3 ± 4.4 Ac	53.3 ± 6.0 ABbc	75 ± 10 ABab	100 ± 0 Aa	5 (0.004–0.006) B	20 (0.013–0.030) A
Fraction 4	18.3 ± 6.0 Bb	20 ± 2.9 Ab	43.3 ± 8.3 ABb	66.7 ± 3.3 ABab	88.3 ± 11.6 Aa	7 (0.005–0.01) B	90 (0.043–0.185) B
Compd1	6.7 ± 4.4 Bc	6.7 ± 4.4 Bc	3.3 ± 3.3 Bc	58.3 ± 8.8 ABb	100 ± 0 Aa	10 (0.008–0.013) B	30 (0.02–0.05) A
Compd 4	1.7 ± 1.7 Bb	8.66 ± 5.0 Bb	–	13.2 ± 7.6 Ba	–	10.2 (0.008–0.017) B	50 (0.02–0.12) A
Azadirachtin^a^	43 ± 12.0 Ab	51.7 ± 10.1 Ab	73.3 ± 14.2 Aab	90 ± 0 ABab	100 ± 0 Aa	1.69 (0.001–0.002) A	10.25 (0.008–0.020) A

*Note*: Means followed by the same upper-case letter within the same column (treatment doses and LSD50/90) and by the same lower-case letter within the same row (treatment doses only) are not significantly different (α = 0.05) after mean separation by Tukey’s HSD test

*Abbreviation*: *SE* standard error

^a^ Positive control

Larval mortality varied with both dose of treatment and exposure time. At 100 ppm, the crude seed extract, fraction 3, aurantio-obtusin (**1)** and azadirachtin elicited the highest larval mortaility, ~ 100% mortality after 24 h of larval exposure to the treatments (Fig. 2). While the bioactivity of the crude seed extract was retained at 10-fold dilution (10 ppm), the bioactivity of fraction 3, aurantio-obtusin and azadirachtin decreased drastically at the same dose to ~40–60% mortality, with azadirachtin eliciting the least larval mortality to ~10%. In general, except for the crude extract and azadirachtin, whereby larval mortality increased with exposure time at low doses (1 and 2.5 ppm), exposure time had minimal effect on the bioactivity of the other treatments at the same doses.

## Discussion

The results of this study show that the crude ethyl acetate extract of *C. tora* is toxic to larvae of the malaria vector *A. gambiae*. Previous studies have shown that the most effective mosquito larvicidal extracts are derived from plants in the families Meliaceae, Asteraceae and Lamiaceae [23]. Bioactivity of these extracts have been attributed to specific classes of chemicals such as limonoids in the Meliaceae, sesquiterpene lactones in the Asteraceae and terpenoids in the Lamiaceae, or different classes of chemicals acting synergistically [19–21, 24]. A recent study documented that the mosquito larvicidal principals in the crude seed extract of *Milletia pinnata*, a plant belonging to the same family Fabaceae as *C. tora*, were derived from various chemical classes including flavonoids, saturated and unsaturated fatty acids [25]. Mosquito larvicidal compounds may act as toxicants, insect growth regulators, anti-microbials against endosymbionts of the larvae, or serve as juvenile hormone blockers in physiological changes such as metamorphosis [26, 27]. Other researchers have shown that a mixture of the same class of compounds in an extract can equally exert the same effect elicited by a blend of compounds derived from different chemical classes. Structural modifications in the basic skeleton of the molecule, can alter bioactivity based on the types and/or positions of substituents in the molecule [19, 20, 28]. Previous researchers have also shown that a key compound in an extract can account for the full bioactivity of an extract [19, 29]. Our chemical analysis of the crude seed extract by LC-qToF-MS revealed predominantly the presence of anthraquinones. As earlier suggested, these compounds may act in combination with each other or with other unidentified classes of chemicals to account for the bioactivity of the crude seed extract. Further research is required to confirm if the different anthraquinones in the crude seed extract of *C. tora* combine with each other or with other classes of chemicals in the extract to explain its bioactivity.

When we fractionated the crude extract, the larvicidal activity decreased, confirming our earlier suggestion that toxicity of the extract was due to a blend of the different compounds. Purification of bioactive fractions led to the isolation of the key anthraquinones, aurantio-obtusin and obtusin. Their bioactivity was similar, with LD_50_ values of 10 ppm, which was four- and six-fold less potent than the crude ethyl acetate extract and the positive control azadirachtin, respectively, and with respect to larval exposure time. The similarity in their bioactivity is not surprising because of the similarity in their structures. Whereas aurantio-obtusin has a hydroxy group at the C-3 position, a methoxy group is located at the same position in obtusin. This suggests that bioactivity of the two different compounds is not dependent on the substituent located at this position, but rather bioactivity may be distributed across the entire molecule, as found for compounds in other studies [19, 22]. Mosquito larvicidal activity of the other structurally different anthraquinones in the extract would help shed light on this suggestion.

**Fig. 3:**
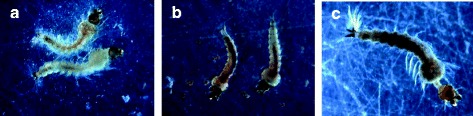
Pictorial representation of dead *A. gambiae* larvae after larvicidal assays. **a** Negative control (DMSO). **b** Crude seed extract of *C. tora*. **c** Azadirachtin (positive control)

The medical importance of anthraquinones isolated from various parts of *Cassia* species has been reviewed recently [30]. Most of the anthraquinones identified have been reported previously to possess a wide range of biological effects, including anti-microbial, anti-viral, anti-inflammatory, anti-tumor, anti-diabetic, antioxidant, and antigenotoxic effects against certain carcinogens, to name a few [13, 31–34]. In the present study, we show that the two major anthraquinones aurantio-obtusin and obtusin are the key mosquito larvicidal compounds identified from fractions 3 and 4 respectively obtained from the seed extract of *C. tora* against larvae of the malaria vector *A. gambiae*. Compared to the crude seed extract and azadirachtin, both aurantio-obtusin and obtusin were moderately effective as mosquito larvicides. When we examined dead larvae exposed to the crude seed extract and azadirachtin (Fig. 3), at the highest dose of 100 ppm, it appeared that larvae were not able to metabolize these specific treatments suggesting that these two treatments might share the same mode of action. On the other hand, the mode of action of fractions 3, 4 and the two individual anthraquinones may be different and worthy of further elucidation. The fact that at 10-fold less than the highest dose (100 ppm) tested, only the crude extract was fasting acting, eliciting the highest larvicidal activity after 24 h compared to that elicited by the other treatments including the positive control azadirachtin suggests that the crude seed extract of *C. tora* is a more favourable option for larval source management than use of its fractions and identified individual key anthraquinones.

## Conclusions

The present study has shown that the crude seed extract and the isolated anthraquinones of the edible leguminous plant *C. tora* possess larvicidal activity against the malaria vector. However, their practical application requires further evaluation in a semi-field and field setting, and on other mosquito species including effect on non-target insects. Because of the tremendous need to reduce and eliminate the burden of vector-borne diseases, the WHO [35] emphasizes a flexible vector control system that supports locally tailored approaches beyond the use of only available effective, evidence-based control interventions. This plant as it is widespread, can be useful in this scenario at a local scale for mosquito population management. This is especially required because of the development of resistance to common larvicides or biological agents including temephos and *Bacillus thuringiensis* impacting on their operational use [3].

## Abbreviations

DMSO: Dimethyl sulfoxide
LC-Qtof-MS: Liquid chromatography–quadruple time of flight–mass spectrometry
PPM: Parts per million
WHO: World Health Organisation
